# An Interactive COVID-19 Vaccine Hesitancy Workshop for Internal Medicine Residents and Medical Students

**DOI:** 10.7759/cureus.27079

**Published:** 2022-07-20

**Authors:** Andrew V Raikhel, Kevin Blau, Katherine Alberty, Jeffrey W Redinger

**Affiliations:** 1 Internal Medicine, VA Puget Sound Health Care System, Seattle, USA; 2 Internal Medicine, University of Washington, Seattle, USA

**Keywords:** student education, internal medicine residents, covid-19 vaccine, medical education & training, vaccine hesitancy, covid 19

## Abstract

Introduction

Since being first identified in December 2019, SARS-CoV-2 has resulted in millions of illnesses and deaths worldwide. Despite the safety and availability of effective vaccines that offer protection from severe COVID-19 disease, a sizable minority of the United States population has reported COVID-19 vaccine hesitancy and remains unvaccinated.

Methods

We developed an interactive workshop for internal medicine residents and medical students in which a framework is utilized to aid the subtyping of COVID-19 vaccine hesitancy. Learners then interactively apply this framework through vaccine counseling in a role-playing exercise.

Results

The workshop increased confidence in the learner's ability to determine the COVID-19 vaccine hesitancy subtype after participation in the workshop (53% preworkshop, 81% postworkshop, p=0.01). The workshop also increased reported confidence in tailoring COVID-19 vaccine counseling after participation in the workshop (60% preworkshop, 90% postworkshop, p=0.005). These gains were also seen when participant learners were compared with nonparticipant learners for both subtyping confidence (81% postworkshop, 26% nonparticipant, p<0.0001) and confidence in providing tailored counseling (90% postworkshop, 60% nonparticipant, p=0.004).

Conclusion

The implementation of our workshop correlated with an increase in the reported trainee confidence related to COVID-19 vaccine counseling. This offers a promising early step in developing educational programs that build trainee skills in this domain. More work is needed to establish robust curricula to support learners in reaching patients who express COVID-19 vaccine hesitancy.

## Introduction

Since being first identified in December 2019, infections from COVID-19 have resulted in millions of illnesses, hospitalizations, and deaths worldwide [1]. A remarkable development to counter these outcomes has been the creation of safe and effective vaccines for COVID-19 [2,3]. Many outreach campaigns have been aimed at persuading unvaccinated individuals to become immunized for COVID-19. Most of this work has been pursued in ambulatory and community settings with significant impact [4]. Despite wide accessibility and robust protection from severe COVID-19 infection, a sizable minority of the population in the United States has reported COVID-19 vaccine hesitancy and remains unvaccinated [5]. Vaccine hesitancy, a longstanding reaction to vaccines, is driven by many interwoven factors, including demographics, accessibility, trust in medical establishments, perception of vaccine safety, and vaccine misinformation, among other factors [6-8]. Previous work has shown that, while challenging, tailored communication offers promising avenues for addressing vaccine hesitancy [9]. Addressing COVID-19 vaccine hesitancy requires physicians to be skilled in having nuanced discussions tailored to each patient's specific concerns. 

Previous educational efforts developed for other vaccine-preventable diseases have utilized a hybrid model combining foundational didactic content and communication frameworks followed by role-playing exercises [10]. These interventions have demonstrated a positive impact on learner confidence and increased the likelihood of learners offering vaccines to patients, thus showing promise for exploration in the COVID-19 setting. Despite this, vaccine training has been underinvested for trainees in adult medicine [11,12]. This has been observed in work showing that Internal Medicine Residents (IMRs) have limited confidence and experience in counseling patients with questions and concerns regarding adult immunization [13]. 

Given the novel and dynamic nature of COVID-19, understanding best practices for addressing COVID-19 vaccine hesitancy requires persistent and continued efforts. This has exposed a need for curricula that fosters communication skills in adult medicine providers on COVID-19 vaccine hesitancy. The severity of the COVID-19 pandemic has impacted our community, and daily life has highlighted the importance of taking every opportunity to engage patients on the importance of COVID-19 immunization. Unvaccinated adult patients admitted to healthcare facilities are a subset that may have received limited previous vaccine hesitancy counseling. This presents a potential opportunity to provide counseling for COVID-19 vaccination to these patients. 

Limited efforts have been developed to address the current deficit in training that IMRs receive related to engaging patients with COVID-19 vaccine hesitancy. To address this issue, we created and implemented a workshop that empowers IMRs with elementary skills to tailor their vaccine hesitancy engagement to the needs of specific patient concerns. This workshop was introduced as a component of a broader initiative to increase the rates of inpatient COVID-19 vaccine administration at our hospital. We hypothesized that once learners were taught and allowed to utilize a framework for navigating COVID-19 vaccine hesitancy, learner confidence regarding COVID-19 counseling would increase.

## Materials and methods

The participants were IMRs in the inpatient medicine ward and consulting teams and 3rd-year medical students rotating on their Internal Medicine clerkship at our hospital. We invited all residents and medical students assigned to inpatient and consult rotations at our hospital to participate. Resident learners rotate at our hospital several times resulting in multiple opportunities for learners to participate in the workshop. There was no prerequisite knowledge or preparatory work required of the learner participants. An in-person facilitator led the workshop. The instructor needed no prior knowledge; however, previous engagement with patients on COVID-19 vaccination was an asset to leading the large group discussions. The workshop lasted approximately 50 minutes in length. Participation was voluntary and took place during a regularly scheduled daily conference, and offered the workshop five times at the beginning of residents' hospital medicine rotation. 

We used a conference room for the workshop. Chairs were divided into groups of three to facilitate learner role play engagement. A PowerPoint presentation was made using a projector. This workshop was also offered once in a remote learning environment without alteration to the workshop structure. Remote teaching of the workshop can be employed provided that the conference platform can facilitate screen sharing and breakout rooms. 

The facilitator started the session with a PowerPoint presentation. The PowerPoint presentation began with an overview of the COVID-19 pandemic, explicitly focusing on current local COVID-19 infections, hospitalizations, and vaccination rates. Up-to-date regional data was obtained from the Johns Hopkins Coronavirus Resource Center [14] and added to the existing PowerPoint slides. 

After this, we shared a framework for understanding several dominant subtypes of COVID-19 vaccine hesitancy. Many of the previously developed vaccine hesitancy models encapsulate the complexity and nuance embodied in decisions regarding vaccination with great granularity [15,16]. While comprehensive, we were concerned that these models are challenging to apply clinically for early learners, given the depth and scale of the included variables. We utilized a framework that employed this complex web of factors in surveying individuals and then mapped the responses into several broader categories of vaccine behavior. The framework subdivided COVID-19 vaccine-hesitant patients into four dominant subtypes: cost-anxious, system distruster, watchful, and COVID-skeptic. We developed this COVID-19 vaccine hesitancy framework from a national telephone and social media survey completed during the current pandemic [17]. In this framework, organized individuals were into psychobehavioral subtypes based on their perceptions regarding several COVID-19 vaccine-related themes, including perceived cost barriers, safety, the personal threat of infection, conspiracy beliefs, and racial fairness in the medical establishment. We recognized that some patients might have elements of more than one subtype that guide their COVID-19 vaccine behavior. To prepare learners for this, we aimed to create a digestible and easily interpretable model to scaffold learners' understanding of COVID-19 vaccine hesitancy. The vaccine hesitancy subtypes mirrored other COVID-19 vaccine hesitancy frameworks generated directly from surveying Veteran populations [18]. 

Definitions of each COVID-19 vaccine-hesitant category were explained to learners: Patients who are cost-anxious often delay seeking care because of perceived time and financial costs associated with vaccination. Patients distrustful of systems feel they are not treated fairly by the medical community, often due to healthcare access and inequity issues. Watchful patients are influenced by social norms and need to see that others in their community have positive experiences with vaccination. Patients who are COVID-skeptics have beliefs about COVID-19 or vaccination, which generally leads to concerns about vaccine safety. For each vaccine-hesitant category, we provided learners with specific patient counseling strategies. For instance, learners were encouraged to avoid debunking erroneous beliefs held by patients in the COVID-skeptic subtype and instead listen to their concerns, acknowledge how they feel, and then explain facts about vaccination while emphasizing vaccination is their personal choice. 

After introducing this COVID-19 vaccine hesitancy framework, the learners applied this knowledge to three role-playing scenarios. Each group self-assigned one learner to be the patient, one to be the physician, and another to be an active observer. Learners were then given five minutes to engage in role-play around each scenario. Learners in the physician role were asked to practice determining the patient subtype presented in each scenario and tailoring their counseling to that specific patient's concerns. After five minutes of role-playing, the scenario was debriefed in a large group. 

The "patient" role was provided with a brief description of the patient's beliefs about COVID-19 vaccination or healthcare systems in general. These descriptions were printed before the start of the workshop and handed out to the learner playing the "patient" before each role-playing round. Each scenario was written to express a specific subtype of COVID-19 vaccine hesitancy. Scenarios were trialed with representative IMR learners before implementation to ensure usability and fidelity to original COVID-19 vaccine hesitancy subtypes. The learner playing the "patient" role was tasked with discussing with the physician the mindset outlined on the scenario card. 

A large group discussion occurred after every scenario that was role-played and started by asking the learners in the physician and observer roles to identify the subtype of vaccine hesitancy in the scenario. Facilitators were instructed to ask learners the following questions to prompt discussion about the counseling that occurred: What went well in the scenario? What was challenging with the scenario? Were any impactful phrases used? Has anybody encountered patients with similar vaccine hesitancy in the hospital or their clinic? How did you navigate these interactions? 

After the debrief of the first scenario, the learners repeated the exercise with the second and third scenarios. Scenarios two and three were handed out to the rotating patient role before the beginning of each round of role play. During each scenario, the roles of patient, provider, and active observer rotated such that after three scenarios, each learner had an opportunity with each role. After completing the third scenario and debriefing, the workshop returned to the PowerPoint slides, which outlined the take-home points. 

The workshop was implemented as a component of a hospital quality improvement effort to increase inpatient COVID-19 vaccination rates. The encompassing quality improvement project gamified COVID-19 vaccine patient engagement with medicine ward teams caring for admitted patients at our hospital. Ward teams tallied administered COVID-19 vaccines and other forms of COVID-19 vaccine engagement as part of this effort. Tallied vaccine tasks were scored points, and winning team members were awarded a certificate at the end of every 4-week rotation. We implemented the vaccine hesitancy workshop during the second plan-do-study-act cycle of the quality improvement cycle. While this educational intervention was implemented as part of a quality improvement effort to increase vaccine engagement, we can implement it independently. 

Statistical analysis

Learners were given a pre-assessment survey before participating in the workshop. The surveyed respondents rated their responses on a 5-point Likert scale (1= strongly disagree, 5 strongly agree). The assessment tool was developed to gauge learner perspective on the importance of engaging patients on their COVID-19 vaccination status while admitted and their confidence and previous experience in having these discussions. The assessment was trialed with representative learners before implementation to ensure clarity and usability. Learners were given the same survey at the end of their inpatient rotation, approximately three weeks after participation in the workshop. Nonparticipating residents were also sent the pre-assessment survey to help delineate differences between equivalent learners who were not involved in the workshop. We compared groups for statistical significance using the Fischer exact test. Responses from all five workshop sessions were analyzed together. This quality improvement project was reviewed jointly by the Human Research Protection Program (HRPP) and Quality, Safety & Value service line at our institution and determined to not constitute human subjects research.

## Results

This workshop was implemented at our hospital as part of the Internal Medicine rotation during the 2021-2022 academic year and offered during five different sessions. Each session had six and eleven learners in attendance, and we had 25 residents and 17 medical students participate in over five sessions. Thirty nonparticipating residents took the pre-assessment survey (table 1). 

**Table 1:** Survey participant and nonparticipant respondents organized by training level

	Participant Learners, N (%)	Nonparticipant Learners, N (%)
Medical Student	17 (40.4%)	NA
Intern (PGY1)	14 (40.4%)	9 (30%)
Senior Resident (PGY2-3)	8 (19.0%)	21 (70%)

**Figure 1:**
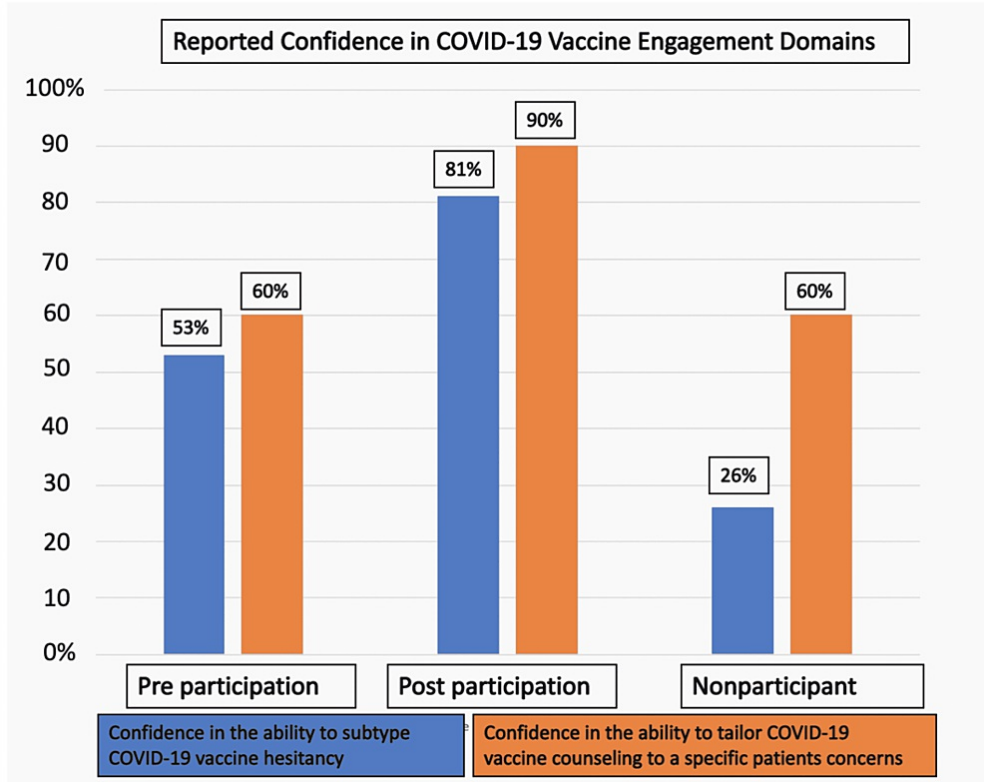
Reported confidence in COVID-19 vaccine engagement domains of learners before and after participation in the workshop, as well as nonparticipant learners.

100% of learners agreed that counseling patients who were unvaccinated for COVID-19 are a critical task to complete during an inpatient admission. We noted an increase in reported confidence in learner ability to determine the COVID-19 vaccine hesitancy subtype after participation in the workshop (53% preworkshop, 81% postworkshop, p=0.01). The postworkshop reported confidence in subtyping was also higher than nonparticipant residents surveyed (81% postworkshop, 26% nonparticipant, p<0.0001). We further noted an increase in reported confidence in tailoring COVID-19 vaccine counseling after participation in the workshop (60% preworkshop, 90% postworkshop, p=0.005). This postworkshop reported confidence in tailoring vaccine counseling was also higher than the nonparticipant learners (90% postworkshop, 60% nonparticipant, p=0.004) (figure 1).

These gains in reported confidence were also observed when the survey data was stratified by resident training level (table 2). Reported confidence in subtyping vaccine hesitancy type was higher amongst participating interns and senior residents compared to nonparticipating trainees (14% nonparticipant intern, 71% participant intern, p=0.01; 33% nonparticipant senior resident, 100% participating senior resident, p=0.002). Reported confidence in tailoring vaccine hesitancy counseling was higher amongst participating interns and senior residents compared to nonparticipating trainees (42% nonparticipant intern, 75% participant intern, p=0.19; 73% nonparticipant senior resident, 100% participating senior resident p=0.14). 

**Table 2:** Participant and nonparticipant reported COVID-19 vaccine counseling confidence organized by training level

	Participant intern, N (%)	Nonparticipant intern, N (%)	P value
Subtyping confidence	10 (71%)	1 (14%)	0.001
Tailoring confidence	11 (75%)	9 (42%)	0.19
	Participant senior resident, N (%)	Nonparticipant senior resident, N (%)	
Subtyping confidence	8 (100%)	7 (33%)	0.002
Tailoring confidence	8 (100%)	15 (73%)	0.14

## Discussion

This is the first curriculum to utilize a vaccine hesitancy framework and role-playing to help learners counsel patients who express COVID-19 vaccine hesitancy. Our workshop increased learner confidence in determining COVID-19 vaccine hesitancy subtypes and tailoring COVID-19 vaccine counseling to specific patients. These gains in learner confidence were reported three weeks after participation in the intervention, demonstrating retention of the workshop's learning objectives. We observed a statistically significant increase in the reported confidence in vaccine hesitancy subtyping compared to pre-implementation survey results and nonparticipating learners. This statistically significant increase was also seen when the vaccine hesitancy subtyping groups were stratified to the intern (PGY1) training level. These results are an encouraging step towards empowering learners in engaging with vaccine-hesitant patients, a crucial step in our response to the COVID-19 pandemic. 

Beyond these increases in learner confidence, we noted the workshop's significant emotional debriefing component. During the large group discussions in each iteration of the workshop, learners shared previous challenging experiences engaging with patients, family, and friends on COVID-19 vaccines. This offered an opportunity for reflection and decompression from these challenging engagements in a safe environment of medical trainee peers. The normalization of this emotional hardship and how the learners processed it was an unplanned yet impactful theme that repeatedly emerged from the significant group discussions. Given the emotional difficulties healthcare workers have faced due to the COVID-19 pandemic, further efforts are needed to provide productive avenues to process these experiences. 

It is important to note that vaccine hesitancy is an incredibly complex, nuanced topic, and patient motivations can rarely be focused on a single element. Because of this, we do not feel this curriculum should be viewed as a definitive or capstone curriculum on COVID-19 vaccine hesitancy. Instead, it can be used as the launching pad whereby learners can develop their COVID-19 vaccine hesitancy counseling skills in their practice with mentored coaching as they discuss these topics with patients. Continued work is needed to build and validate ongoing resources for advanced adult medicine practitioners for COVID-19 vaccine hesitancy. Though we aimed our efforts at medical trainees, our workshop may be useful as a model of curriculum that could be extrapolated to non-clinicians who engage with vaccine-hesitant populations, including teachers of school-age children and school staff. As in our workshop, concentrating these efforts on communication strategies may prove helpful in multiple public health settings. 

Interestingly, the workshop introduction did correlate with an increase in the number of COVID-19 vaccines administered at our hospital in the inpatient setting. As was mentioned, the vaccine hesitancy workshop was implemented as part of a broader quality improvement effort at our hospital to increase the rate of inpatient COVID-19 vaccine administration [19]. Many factors likely influenced the rate of COVID-19 vaccine administration at our hospital. These include other elements of our quality improvement project and external factors such as modulating guidelines from the Centers for Disease Control that increased vaccine eligibility. While we cannot conclude that the workshop resulted in an increased rate of COVID-19 vaccination, this correlation is promising and points to the potential future work can have in the efforts to increase vaccination acceptance amounts adult patients. 

This workshop had several limitations. Using a subtyping framework for vaccine hesitancy limits the innumerable nuances that inform patient decisions regarding vaccine hesitancy. The scope of this curriculum was limited to introducing a manner of tailoring COVID-19 vaccine counseling. Patients may exhibit elements of multiple subtypes or may not fit into this framework at all. The decision to use this framework was considered a useful initial scaffolding structure for learners to parse differences in patient motivation into a workable framework. As such, we recognize that we will need continued practice in determining the individual motivations of patients for learners to continue their vaccine counseling growth. 

The learner cohort that participated in the workshop was small, and this may impact the reported change that the workshop had on learner confidence. Additionally, COVID-19 vaccine beliefs will likely transform and develop as our relationship with the disease evolves. This may result in the need for renewal and revisions of the vaccine hesitancy subtyping framework and associated patient scenarios. Additionally, it is unclear at this time if there will be any long-term impacts on learner COIVD-19 vaccine counseling. Further work is needed to assess this. 

We would have liked to compare participating medical students with nonparticipating medical students similarly to what was assessed with interns and residents. However, this was not possible due to challenges related to surveying this cohort. We were also unable to compare the effectiveness of the workshop when comparing the in-person versus remote learners due to the limited number of learners who participated in the workshop in the remote setting. 

## Conclusions

As the COVID-19 response continues, the development of robust communication techniques must reach patients who express COVID-19 vaccine hesitancy continues. Our experiences have shown that many patients have deeply held barriers to vaccination, but these barriers can modulate in intensity and character. We must develop ongoing pedagogical training efforts to train various healthcare workers with the tools to counter COVID-19 vaccine hesitancy. With continued efforts and tenacity, we have an opportunity for future medical providers to decrease the prevalence of COVID-19 vaccine hesitancy. 
